# Effect of sancai powder on glacemic variability of type 1 diabetes in China: A protocol for systematic review and meta-analysis

**DOI:** 10.1097/MD.0000000000020772

**Published:** 2020-08-21

**Authors:** Weiwei Yu, Dongqi Zhou, Li Zhang, Chen Rumeng, Peishuai Zhang, Lisha Sun, Ziping Gao

**Affiliations:** a Hospital of Chengdu University of Traditional Chinese Medicine, Chengdu, Sichuan Province, China; b Chengdu University of Traditional Chinese Medicine, Chengdu, Sichuan Province, China.

**Keywords:** glacemic variability, saicai powder, traditional Chinese medicine, type 1 diabetes

## Abstract

**Background:**

Type 1 diabetes mellitus (T1DM) is a chronic, immune-mediated disease characterized by the destruction of insulin producing cells and persistent hyperglycemia. At present, the drugs for type 1 diabetes mellitus can reduce blood glucose rapidly and effectively, but there are risks of hypoglycemia, large fluctuation of blood glucose, and chronic complications. Related research found that compared with continuous hyperglycemia, blood glucose fluctuations are more harmful to the chronic complications of diabetes. Blood glucose variation is closely related to the occurrence and development of chronic complications of diabetes. Sancai powder (SC) is made on the basis of 3 ancient Chinese medicine formulas, which has the effect of lowering blood glucose. There have been reports on the clinical study of SC in the treatment of diabetic patients, but there is no systematic evaluation of SC in the treatment of type 1 diabetes, so it is necessary to summarize and evaluate the existing evidence.

**Methods and analysis:**

This study will be conducted according to Preferred Reporting Items for Systematic Reviews and Meta-analysis Protocols. We will search 3 English databases and 4 Chinese databases. Two methodologically trained researchers will read titles, abstracts, and full texts, and independently select eligible literature based on inclusion and exclusion criteria. After assessing the risk of bias and extracting data, we will conduct a meta-analysis of the results, including: standard deviation of blood glucose level, coefficient of variation, mean blood glucose, postprandial blood glucose fluctuation, hypoglycemia index, glycated hemoglobin, overall impact rate, and adverse effects. The heterogeneity of the data will be tested by Cochrane x2 and I2. Based on reliable subgroup effect guidance, we established 3 hypotheses for subgroup analysis: disease status at baseline, duration of intervention, and type of concomitant medication. Sensitivity analysis will be carried out to assess the stability of the results. The publication bias assessment will then be performed by funnel plot analysis and Egger test. Finally, we will use the “grading, evaluation, development and evaluation of recommendations” system to assess the quality of evidence.

**Results:**

The results of this systematic review and meta-analysis will be published in a peer-reviewed journal.

**Conclusion:**

In our study, the evidence of SC in the treatment of reducing blood sugar fluctuation in type 1 diabetes will be comprehensively summarized and carefully evaluated. It will provide more options for clinical treatment of the disease.

**INPLASY registration number:**

INPLASY202050052

## 1 Introduction

Type 1 diabetes mellitus (T1DM) is an autoimmune disease, which mainly occurs in children and adolescents, and exists in all ages. The main causes of the disease are genetic factors, environmental factors, autoimmune response, virus infection, and drug exposure. In this way, the immune response of the body is disturbed, and the autoantigen is activated, which destroys the B cell of the islet of Langerhans, and the insulin of the body is absolutely insufficient, leading to diseases. It is characterized by rapid onset, long duration, multiple complications, as well as thirst and polyuria.^[1]^ T1DM accounts for approximately 90% of total diabetes in children and adolescents in Western countries and is the most common form of childhood diabetes in most parts of the world.^[2]^ Although China's type 1 diabetes registration study has confirmed that China is one of the countries with the lowest incidence of type 1 diabetes in the world, due to the large population in China, the actual number of type 1 diabetes patients is not small. The study shows that there are at least 13,000 new cases of diabetes every year in China, of which 4271 are type 1 diabetes patients.^[3]^ According to data from the International Diabetes Federation world diabetes map 2017, the number of newly diagnosed children with type 1 diabetes in China ranks fourth in the world, and the top 3 are followed by the United States (14,700), India (11,300), and Brazil (7600).^[4]^ Treatment of T1DM puts a heavy burden on the government's finances.

In addition to controlling high blood sugar, patients with type 1 diabetes should pay attention to blood sugar fluctuations. The harm of blood sugar fluctuations to chronic complications of diabetes is even greater than that of persistent high blood sugar.^[5,6]^ Blood sugar fluctuations are closely related to the occurrence and development of chronic complications of diabetes.^[7–10]^ Related studies have shown that insulin resistance, oxidative stress, and inflammatory factors can cause blood sugar instability in diabetic patients.^[11,12]^ This increases the risk of various complications of diabetes. The main treatment methods of TIDM include islet/pancreatic cell transplantation, insulin injection, weight loss, stem cell therapy, and immune intervention. These therapies have their own limitations. Due to the presence of autoimmune reactions, islet/pancreatic cell transplantation cannot fundamentally solve the problem and is expensive. Although insulin injections are convenient and fast, many patients have difficulty persisting with daily injections,^[13]^ which can easily cause hypoglycemia and exacerbate blood sugar fluctuations. Weight loss therapy is not suitable for non-obese type 1 diabetic patients. Stem cell therapy and immune intervention therapy have great potential, but are not yet mature, and further research is needed,^[14,15]^ so high-quality research is urgently needed. This study will systematically collect clinical studies of sancai powder (SC) treatment of T1DM, and conduct a systematic review and meta-analysis to provide a reliable basis for clinical practice.

SC comes from the Chinese Medicine ancient book “Wen Bin Tiao Bian”,which is composed of 6 traditional Chinese medicines (TCMs) such as Tian Dong, Raw Dihuang, Ginseng, Coptis, Cinnamon, and Dark Plum. Designed to balance the yin and yang of the human body. SC has clear efficacy in treating diabetes. Modern pharmacological studies have shown that the 6 herbs in its group have hypoglycemic effects. In addition, raw dihuang can inhibit the production of inflammatory factors, and ginseng has both anti-oxidant and anti-inflammatory effects. Coptis can improve insulin action on target cells and relieve insulin resistance. Cinnamon can increase insulin sensitivity and reduce insulin resistance. In addition to reducing blood sugar, dark Plum also has antioxidant effects. SC strictly follows the theory of compatibility of TCM prescriptions and SC in multiple mechanisms, multi-targeted way to lower blood sugar levels, relieve glycemic excursions. SC has been widely used in the prevention and treatment of diabetes in China, but there is currently no meta-analysis on SC to improve blood glucose fluctuations in type 1 diabetes. Therefore, the urgent need for high-quality research. In this study, we will systematically collect SC clinical study of type 1 diabetes treatment, and a systematic review and meta-analysis to provide reliable evidence for clinical practice.

## 2 Objectives

**To investigate:**

1. Efficacy and safety of SC on Chinese patients with type 1 diabetes.
2. Whether SC can effectively reduce the blood sugar variability of patients with type 1 diabetes in China.

## 3 Methods and analysis

### 3.1 Study registration

The protocol for this systematic review was registered on INPLASY(INPLASY registration number:INPLASY202050052, DOI:10.37766/inplasy2020.5.0052)and is available in full on https://inplasy.com/inplasy-2020-5-0052/. The article will be reported in accordance with the Preferred Reporting Items for Systematic Review and Meta-Analysis Protocols checklist.^[16]^

### 3.2 Inclusion criteria

Those randomized controlled trials that treated T1DM with SC in combination with placebo or conventional treatments will be included in our study. Conventional treatments include oral hypoglycemic medications and insulin injections. Of course, insulin pumps are among the routine treatments. TIDM diagnosis based on World Health Organization (1999) criteria. In order to obtain as many references as possible and reduce bias, we do not have any restrictions on factors such as age, gender, and duration of illness.

### 3.3 Exclusion criteria

Studies that met the following criteria will be excluded for the meta-analysis:

1. increased blood sugar is not due to T1DM being excluded.
2. In some studies, the authors uses SC and other medicines together in experimental group, since SC is not the main intervention in these studies, these studies will be excluded.
3. Studies whose outcomes are not directly related to reduce glycemic excursions or lower blood sugar.
4. Repeatedly published articles.

### 3.4 Outcomes

#### 3.4.1 Primary outcomes

Standard deviation of blood glucose level and coefficient of variation .

#### 3.4.2 Secondary outcomes

1. MBG, PPG, hypoglycemia index, glycated hemoglobin.
2. Total effective rate; the judgement of effectiveness takes into account the improvement of examination indicators and the relief of clinical symptoms.
3. Hyperglycemia or hypoglycemia that may occur during the study.
4. Have any adverse effects of medication throughout the process.

### 3.5 Study search

We will search 4 Chinese databases, including articles knowledge service platform, VIP information resource integration Service platform , the China National Knowledge Infrastructure database, China Biomedical CD (Sino Med), as well as 3 English databases, including Embase, PubMed, Cochrane Library Controlled Trials Register, Chinese, and English restricted languages. In addition, to identify unpublished studies or other relevant documents, we will also search Google scholar, Baidu scholars. Last but not least, the Chinese Clinical Trials Registry, and ClinicalTrials.gov will also be searched. A manual search will be performed in the Chengdu University of TCM library. We will use a search strategy that combines MeSH words with free words. Two authors will search and screen all the citations independently. The process of search is presented in the following Table 1.

**Table 1:**
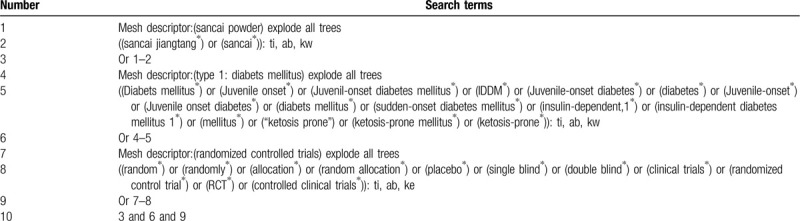
Example of Cochrane search strategy.

### 3.6 Study selection

**Figure 1:**
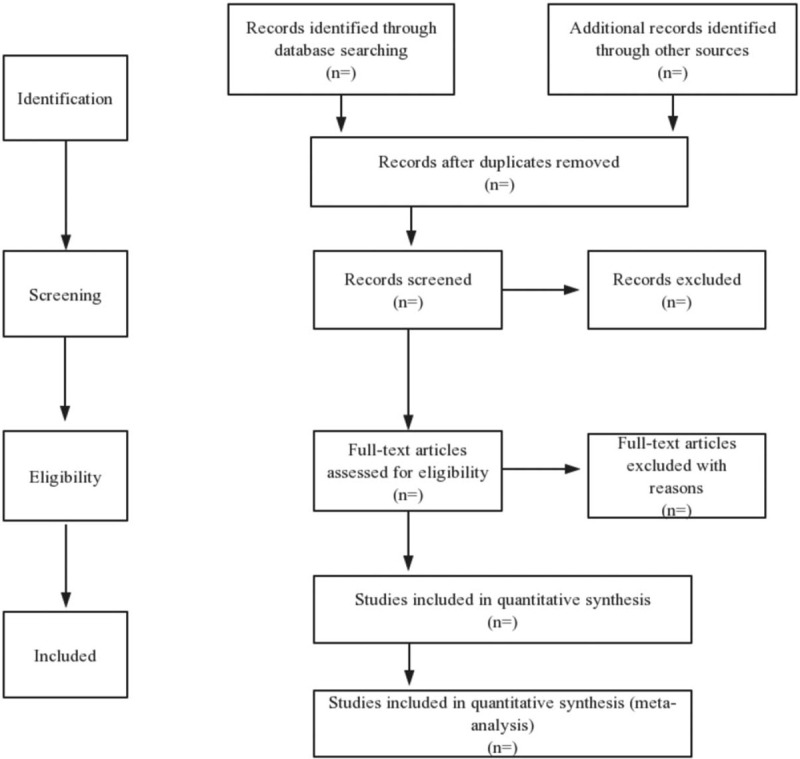
Study selection flow chart.

First, the electronic references downloaded from the above database will be managed by us in the Mac version of endnote X7 (The Thomson Corporation and Reuters Group PLC, Toronto, Canada and New York). In the second part, 2 researchers trained in methodology read titles, citations and abstracts, and selected citations according to inclusion and exclusion criteria. By reading the full text, they will further determine those articles that meet the conditions. In case of any difference, they will make a final decision by consensus. A flowchart has been drawn to show the process of study selection (Fig. 1).

### 3.7 Data extraction

We will extract the data of these qualified articles into Microsoft Excel. For each study, the following data will be extracted: first author of the article, year of publication, country, intervention in the experimental group, intervention in the control group, number of patients in each group, age, and gender of patients, BMI, lifestyle, course of disease, Time of illness, results, and safety data. If there is not enough data in the study, we will contact the corresponding author for more detailed data. When the details of the method are not mentioned in the paper to be extracted, we will contact to provide further instructions.

### 3.8 Risk of bias assessment

The 2 reviewers will use a mature and reliable way to assess the risk of bias in the study: the Cochrane Collaboration tool. In this tool, 7 items were used to evaluate the risk of experimental bias: random sequence generation (selection bias), assignment concealment (selection bias), blindness of participants and personnel (performance bias), blind (detection) bias of result evaluation, incomplete result data (loss bias), selective reporting (reporting bias), and other biases. Each project is divided into “low risk”, “high risk” or “unclear risk”.^[17]^ Two reviewers will independently assess the risk of bias and any differences will be resolved through discussion by all reviewers.

## 4 Data analysis

Reference management will be completed by MAC's endnote X7 (Thomson Reuters), while Review Manager 5.3 software (International Cochrane Collaboration: Cochrane Systematic Reviews, Nordic Cochrane Center) and MAC's stata14.0 software (Statacorp, Texas) will be used to create forest maps and perform group analysis and sensitivity analysis. For continuous variables, the effect is expressed as mean difference and 95% confidence interval. For binary variables, the impact will be expressed as a risk ratio and a 95% confidence interval and will be calculated using the Mantel–Haenszel (MH) method. The heterogeneity of the data will be studied by Cochrane x2 and I2 tests.^[18]^ When *P* < .05 and I2> 50%, statistical heterogeneity is considered important. If *P* > .05 and I2 <50%, the included studies are homogeneous and the difference between the 2 is negligible. If there is significant heterogeneity, the random effect model is used to aggregate the data; if there is no significant heterogeneity, the fixed effect model is used. If a large amount of heterogeneity does not allow quantitative synthesis, the results will be presented in the form of tables and charts.

## 5 Investigation of heterogeneity

When there is great heterogeneity between studies, we will conduct subgroup analysis and meta-regression to explore heterogeneity. The patient's condition at baseline, the type of medication used during treatment, and the duration of intervention are the 3 hypotheses we established for the subgroup analysis.^[19]^ We will perform subgroup analysis based on these subgroup hypotheses. Then, we will evaluate the credibility of the subgroup analysis according to reliable subgroup analysis guidelines.^[20]^ If there is enough research, then meta-regression will be conducted to further explore the source of heterogeneity.

## 6 Sensitivity analysis

The purpose of conducting sensitivity analysis is to ensure the stability of the research results. Each study included in the analysis will be excluded one by one, and then re-analyze and summarize the data, and compare the difference between the regained effect and the original effect. In this way, the impact of a single study on the overall results and whether the results are reliable will be assessed.

## 7 Publication bias assessment

When there are more than 10 studies in this analysis, the publication bias will be evaluated in the form of funnel chart analysis, and the statistical survey will be conducted by the method of Egger test.^[21,22]^ In the case of *P* < .05,we think there is a publication bias.

## 8 Summary of finding tables

At this stage, we will use a tool that is widely used in quality assessment: Grading of Recommendations Assessment, Development, and Evaluate system to summary finding tables for each out comes.^[23]^ Finally, the evidence will be shown from 5 domains: certainty assessment, number of patients, effect, certainty, and importance. In the Grading of Recommendations Assessment, Development, and Evaluate system system, the quality of evidence can be defined as “high”, “moderate”, “low”, and “very low”.

## 9 Patient and public involvement

There are no patients and the public in this study

## 10 Ethics and dissemination

This meta-analysis does not require moral approval. The existing research evidence of SC will be comprehensively evaluated in our research. Through this strategy, we will provide clinicians with more evidence-based medical support for SC treatment of TIDM. Finally, our results will be published in peer-reviewed journals.

## 11 Discussion

T1DM is a disease should not be underestimated, as the disease progresses, patients often face the risk of various complications of diabetes. In addition, T1DM has been a huge burden on national health care.^[24–27]^ With the increasing application of SC in clinical and a series of clinical studies have been carried out about it. We should systematically and comprehensively summarize its existing clinical evidence. In this study, we will conduct this systematic review and meta-analysis to provide more evidence-based medical support for clinical use of SC.

Although SC has been widely used in the treatment of diabetes in China, this is the first systematic review and meta-analysis of SC in the treatment of type 1 diabetes. The results of the study may help us develop a better way to reduce blood sugar fluctuations in patients with type 1 diabetes, thereby providing patients and clinicians with more choices. The limitation of this analysis is that the number of studies that meet our screening criteria is not many, but in order to obtain as many studies as possible, we do not have any restrictions on factors such as age, gender, and duration of illness.

In this study, we will conduct subgroup analysis and regression analysis in order to obtain more accurate and reliable conclusions to explore the possible heterogeneity between studies. In order to avoid meaningless post-analysis, we will conduct a subgroup analysis based on a predetermined set of hypotheses to assess reliability standards. Finally, in order to provide better guidance for clinical use, we will classify the available evidence.

## Author contributions

**Conceptualization:** Weiwei Yu, Dongqi Zhou, Li Zhang.

**Data curation:** Dongqi Zhou, Li Zhang.

**Formal analysis:** Weiwei Yu, Rumeng Chen.

**Funding acquisition:** Ziping Gao.

**Investigation:** Dongqi Zhou.

**Methodology:** Peishuai Zhang, Rumeng Chen.

**Project administration:** Ziping Gao.

**Resources:** Lisha Sun.

**Software:** Peishuai Zhang, Weiwei Yu

**Supervision:** Rumeng Chen.

**Writing – original draft:** Weiwei Yu, Dongqi Zhou, Li Zhang.

**Writing – review & editing:** Weiwei Yu, Dongqi Zhou, Li Zhang.
